# Posterior Hox gene reduction in an arthropod: *Ultrabithorax* and *Abdominal-B* are expressed in a single segment in the mite *Archegozetes longisetosus*

**DOI:** 10.1186/2041-9139-4-23

**Published:** 2013-08-30

**Authors:** Austen A Barnett, Richard H Thomas

**Affiliations:** 1 Department of Zoology, Southern Illinois University, 1125 Lincoln Drive, Carbondale, IL 62901, USA

**Keywords:** Hox, *Ultrabithorax*, *Abdominal-B*, *Patched*, Segment-polarity, Arthropod, Chelicerate, Opisthosoma, Acari, Acariformes

## Abstract

**Background:**

Hox genes encode transcription factors that have an ancestral role in all bilaterian animals in specifying regions along the antero-posterior axis. In arthropods (insects, crustaceans, myriapods and chelicerates), Hox genes function to specify segmental identity, and changes in Hox gene expression domains in different segments have been causal to the evolution of novel arthropod morphologies. Despite this, the roles of Hox genes in arthropods that have secondarily lost or reduced their segmental composition have been relatively unexplored. Recent data suggest that acariform mites have a reduced segmental component of their posterior body tagma, the opisthosoma, in that only two segments are patterned during embryogenesis. This is in contrast to the observation that in many extinct and extant chelicerates (that is, horseshoe crabs, scorpions, spiders and harvestmen) the opisthosoma is comprised of ten or more segments. To explore the role of Hox genes in this reduced body region, we followed the expression of the posterior-patterning Hox genes *Ultrabithorax* (*Ubx*) and *Abdominal-B* (*Abd-B*), as well as the segment polarity genes *patched* (*ptc*) and *engrailed* (*en*), in the oribatid mite *Archegozetes longisetosus*.

**Results:**

We find that the expression patterns of *ptc* are in agreement with previous reports of a reduced mite opisthosoma. In comparison to the *ptc* and *en* expression patterns, we find that *Ubx* and *Abd-B* are expressed in a single segment in *A. longisetosus*, the second opisthosomal segment. *Abd-B* is initially expressed more posteriorly than *Ubx*, that is, into the unsegmented telson; however, this domain clears in subsequent stages where it remains in the second opisthosomal segment.

**Conclusions:**

Our findings suggest that *Ubx* and *Abd-B* are expressed in a single segment in the opisthosoma. This is a novel observation, in that these genes are expressed in several segments in all studied arthropods. These data imply that a reduction in opisthosomal segmentation may be tied to a dramatically reduced Hox gene input in the opisthosoma.

## Background

Hox genes are highly conserved transcription factor-encoding genes that regulate a large suite of transcriptional targets in all bilaterian animals [1,2]. The conserved role of each Hox gene in specifying distinct body regions along the antero-posterior axis has caused this set of genes to be targets of evolutionary change throughout animal evolution [1,3-6]. Changes in Hox gene function and expression domains have been shown to have led to a wide array of morphological novelties, for example, the limbless bodies of snakes [7], the evolution of the tetrapod limb [8] and the repression of limbs in the insect abdomen [9]. Despite the general observation that changes in Hox gene expression domains correlate with the generation of new morphologies, a relatively less explored phenomenon is how Hox genes are expressed and utilized in body regions that have been secondarily reduced.

The arthropods, including insects, crustaceans, myriapods and chelicerates (arachnids and horseshoe crabs) display a wide degree of morphological variation on their relatively modular and segmented body plan. The origin of this morphological diversity has been due, in large part, to changes in Hox expression domains and targets throughout their evolution [6,10-12]. In the arthropods, Hox genes act to specify the distinct identities of the developing body segments (for example, head versus abdominal). The relationship of Hox genes to the developing segments is further exemplified in the well-studied model arthropod, *Drosophila melanogaster*. In *D. melanogaster*, segments are established via the partitioning of the blastoderm into discrete segmental units by the activation of the gap genes, which subsequently activate the pair-rule and segment polarity genes. The gap and pair-rule gene expression domains are then used to establish the Hox gene domains within each segment [13,14]. It has also been shown that segment polarity genes directly interact with Hox genes to elicit segmental identity in the *D. melanogaster* abdomen [15].

The body plan of chelicerate arthropods is comprised of two main body regions, the anterior prosoma and the posterior opisthosoma. The segments of the prosoma in chelicerates comprise the chelicerae, pedipalps, and four pairs of walking legs. The opisthosoma is more variable and contains the segments bearing the book gills in horseshoe crabs, the chemosensory pectines in scorpions and the book lungs and spinnerets in spiders. Contrary to the morphological variation of the opisthosoma seen throughout many chelicerate groups, the Hox gene *Ultrabithorax* (*Ubx*) has been shown to have a conserved early expression boundary in the second opisthosomal, or genital, segment [16-18]. The expression of the Hox gene *abdominal-A* (*abd-A*) is expressed in more posterior opisthosomal segments in chelicerates, having an anterior boundary in the third opisthosomal segment, which overlaps with *Ubx* expression. The most terminally expressed Hox gene in the chelicerate opisthosoma is *Abdominal-B* (*Abd-B*), which usually overlaps with the expression of *Ubx* and *abd-A* in the posterior opisthosomal segments [16,18-21].

Previously, we have shown that the mite *Archegozetes longisetosus* patterns only two segments in the opisthosoma via the expression of orthologues of the segment polarity genes *hedgehog* (*hh*) and *engrailed* (*en*) [22], indicating a large degree of segmental fusion or loss in comparison to the ancestral chelicerate opisthosoma, which was likely comprised of twelve segments [23]. To determine whether a reduction in posterior segmentation in *A. longisetosus* resulted in changes in Hox gene utilization in the mite opisthosoma, the expression patterns of the *A. longisetosus* orthologues of *Ubx* and *Abd-B* (*Al-Ubx* and *Al-Abd-B*, respectively) were followed. Also, the expression patterns of the segmentation gene *patched* (*ptc*), which encodes an Hh receptor in all other arthropods studied [24-26], were followed to determine if the *Al-hh* and *Al-en* expression patterns were unique, or if *A. longisetosus* truly pattern only two segments. The results of this study suggest that *A. longisetosus* does only pattern two segments in the opisthosoma during embryonic development, and also that *Al-Ubx* and *Al-Abd-B* are both only expressed in the same single segment, a novel observation for any arthropod studied thus far. These data, in conjunction with the observation that acariform mites have lost an *abd-A* orthologue [22,27], suggest that Hox gene input in the mite opisthosoma has been reduced either as a cause of or a consequence of segmental reduction.

## Methods

### *Archegozetes longisetosus* cultures

Mites were reared on a plaster-of-Paris/charcoal substrate in plastic jars to maintain appropriate humidity. Wood chips were added to the jars to promote oviposition. Mites were fed with brewer’s yeast. No ethical approval was needed as *A. longisetosus* is not subject to any animal care regulations.

### Embryo fixation and staining

To collect early-stage embryos (that is, germ band and early segmentation stage), adults were dissected in 1X PBS using a sharpened tungsten needle and sharp forceps. Laid late-stage embryos (that is, post-germ band stage) were collected from the culture chambers with a needle. Embryos of all stages were pooled and dechorionated in 50% bleach for one minute. Fixation occurred in an *n*-heptane solution over 4% formaldehyde in PBS for 45 minutes. Embryos were devittelinized by placing them into an *n*-heptane solution chilled on dry ice, subsequently adding room temperature methanol and then shaking vigorously for one minute to rupture the membrane. Embryos were rehydrated in graded methanol/PBS solutions, and placed in PBS with 0.1 μg/mL 4′,6-diamidino-2-phenylindole (DAPI) for one minute in the dark. A detailed protocol is available from the authors.

### Gene cloning and identification

cDNA was constructed from *A. longisetosus* total embryonic RNA using the SMARTer RACE cDNA Kit (Clontech, Madison, WI, USA). All gene fragments were amplified using this cDNA as a template in rapid amplification of cDNA ends (RACE) PCR reactions. The *A. longisetosus* orthologue of the segment-polarity gene *patched* (*ptc*) was cloned by using the primer *Arlo.ptc.GSP2.1* (GTGTGTGCATTCTTGGCGGCAGCAATTATTCC) in a 3′ RACE reaction. The resulting fragment was subsequently used in a nested 3′ RACE reaction using the primer *N.Arlo.ptc.GSP2.1* (AGGTGTTTTGCTCTTCAGGCTGCAATTCTC). Both of these primers were designed against a fragment retrieved from an expressed sequence tag screen. The resulting 2,976 bp sequence consists of a large 1,816 5′ UTR, a 981 bp coding sequence and a 179 bp 3′ UTR (GenBank: KF155150). The coding sequence encodes a 326 amino acid protein, which contains the diagnostic Eukaryotic Sterol Transporter (EST) family domain [see Additional file 1: Figure S1A]. *Al-en* was cloned and sequenced as described in [22].

The full-length mRNA sequence of the *A. longisetosus Ultrabithorax* orthologue (*Al-Ubx*) was retrieved using both 3′ and 5′ RACE reactions. For the 3′ RACE reactions, the primer *Ubx.Rtry.GSP2* (GCTGCAGCTGAAGCACATCAGGCCTACC) was used in an initial RACE PCR reaction. The resulting product was used in a subsequent nested 3′ RACE reaction using the primer *N.Ubx.Rtry.GSP2* (CTTTACGACGGAGCGACCAGTCAAGCAT). For the initial 5′ RACE reaction, the primer *Ubx.Rtry.GSP1* (CGCCTGTGCCTGTCTCTCCTGTTCGTTT) was used. The resulting product was used in a subsequent nested RACE PCR using the primer *N.Ubx.Rtry.GSP1* (CGACCTCTTCGACGCAGACCGTTGGCAC). All four of the aforementioned primers were designed using the deduced *Al-Ubx* coding sequences of the *A. longisetosus* Hox cluster (unpublished data, we have dense coverage sequence for the Hox cluster region relevant to this paper). This single *Ubx* orthologue was the only one retrieved, and matches the genomic sequence in the cluster, thus indicating *A. longisetosus* likely has only one *Ubx* orthologue.

The resulting 3′ and 5′ nested RACE PCR reactions were cloned into the pGEM T Easy vector and sequenced. The resulting sequences were assembled using PHRAP to construct the full-length sequence of the *Al-Ubx* mRNA (GenBank: KF155151). The 1,759 bp *AlUbx* mRNA sequence consists of a 134 bp 5′ UTR, a 816 bp coding sequence, and a 809 bp 3′ UTR. The deduced amino acid sequence of *Al-Ubx* has a typical *Ubx* homeodomain and the diagnostic C-terminal UbdA motif [see Additional file 1: Figure S1B].

Fragments of the *A. longisetosus Abd-B* orthologue (*Al-Abd-B*) were cloned from embryonic cDNA using primers developed from genomic Hox cluster sequences. The primers used were *AbdB.Rtry.Gsp1* (TAGCCTGTGGAGCACCGGTCCATTCCAG) in the 5′ RACE reaction and the primer *AbdB.Rtry.Gsp2* (GGCCAAACACTCCATATCTCAGCAAAGCGG) in the 3′ RACE reaction. The resulting fragments were used in subsequent nested RACE PCR reactions, using the primers *N.AbdB.Rtry.Gsp1* (GGTGAGTAGTTGCACCAGGCCGCTGCCG) and *N.AbdB.Rtry.Gsp2* (CAGCGGCCTGGTGCAACTACTCACCATA) in the 5′ and 3′ reactions, respectively. These primers are overlapping reverse-compliments of one another. The resulting fragments were cloned into the pGEM T Easy plasmid and sequenced. This single *Abd-B* orthologue was the only one retrieved, and matches the genomic sequence in the cluster, thus indicating *A. longisetosus* likely has only one *Abd-B* orthologue.

The resulting sequences were assembled using PHRAP to construct the full-length sequence of the *Al-Abd-B* mRNA (GenBank: KF155152). The overlapping 3′ and 5′ RACE Abd-B products resulted in a 2,323 bp mRNA, consisting of a 227 bp 5′ UTR, a 1,191 bp coding sequence, and a 905 bp 3′ UTR. The deduced amino acid sequence of *Al-Abd-B* consists of a typical *Abd-B* homeodomain [see Additional file 1: Figure S1C].

## Results

### *patched (Al-Ptc)* expression

**Figure 1:**
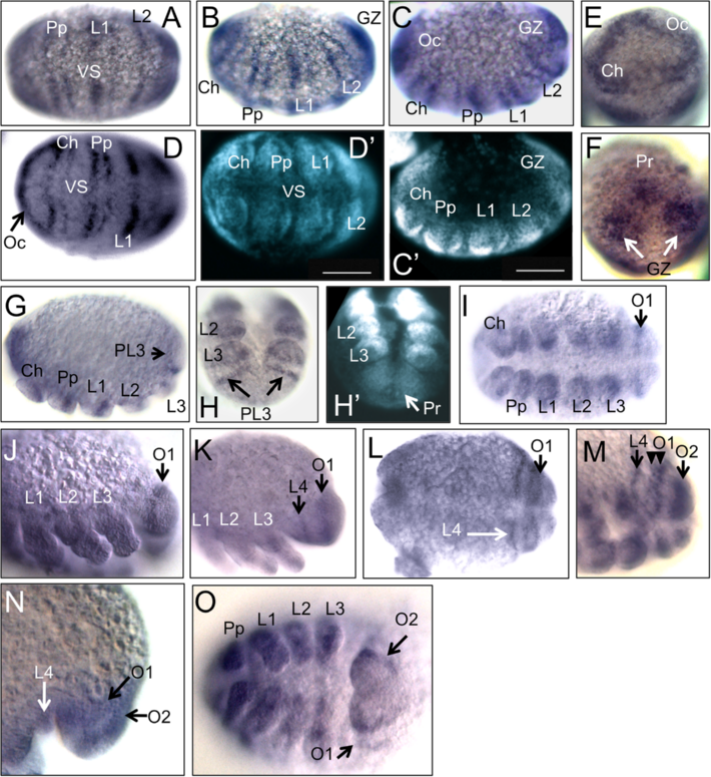
***Al-patched*(*Al-ptc*) expression.A**-**D´**, **G**, **I**, and **J**-**O** are oriented with the anterior pole towards the left of the page. **(A)** Ventral *Al-ptc* expression in an early embryo with *Al-ptc* in the Pp and L1-L2. **(B)** Ventral-lateral view of the same embryo showing double *Al-ptc* in the Ch and the GZ. **(C)** Lateral view of the embryo in **A** and **B**. **(C´)** DAPI image of the embryo in **C**. **(D)** Anterior view of the same embryo showing *Al-ptc* in the Oc. **(D´)** DAPI image of the embryo in **D**. **(E)** Anterior view of the same embryo, showing *Al-ptc* in the Oc. **(F)** The posterior end of the same embryo showing *Al-ptc* in the Pr and the GZ. **(G)** Lateral view of a late embryo showing stripes of *Al-ptc* in the prosoma with a stripe of expression in the PL3. **(H)** Posterior view of the embryo in **G**. **(H´)** DAPI image of the embryo in **H**. **(I)** Ventral view of an embryo showing *Al-ptc* in O1. **(J)** Ventral-lateral view of an embryo showing *Al-ptc* in O1. **(K)** Lateral view of a later-stage embryo showing *Al-ptc* in O1 and in the L4. **(L)** Ventral view of a later-stage embryo showing the split stripe of *Al-ptc* in O1. **(M)** Ventral-lateral view of a later embryo showing the split stripe of *Al-ptc* in O1 (arrowheads), L4 and O2. **(N)** Lateral view of the opisthosoma of a late embryo showing *Al-ptc* in O1 and O2. **(O)** Ventral view of the embryo in **N**. Scale bars in **C**´ and **D**´ are 50 μm. Ch, chelicerae; DAPI, 4′,6-diamidino-2-phenylindole; GZ, growth zone; L1-L3, first through third walking legs; L4, fourth walking leg anlagen; O1-O2, first and second opisthosomal segment; Oc, ocular segment; PL3, posterior portion of the L3 segment; Pp, pedipalps; Pr, proctodaeum; VS, ventral sulcus.

The earliest observed expression of *Al-ptc* was in the early germ band stage (Figure 1A-E), in the prosomal segments that will eventually bear the chelicerae, pedipalps and the first two pairs of walking legs. The segments of the third and fourth pair of walking legs have not been formed at this stage. In all studied arthropods, *ptc* is initially expressed in a single, broad stripe in each segment followed by the splitting of this stripe into two stripes of expression in more mature segments, in which the expression of the segment-polarity gene *en* is situated in between these two stripes [24-26]. The early expression patterns of *Al-ptc* show this double-striped pattern, by which an anterior stripe is expressed in the middle of the developing segment, and a posterior stripe is expressed just posterior to the segmental boundary (Figure 1A-D´; see [22] for early prosomal *Al-en* expression). As this stage is the earliest that was observed, it is unclear whether these prosomal *Al-ptc* expression patterns began as single broad stripes. *Al-ptc* expression was also expressed in a continuous stripe anterior to the two cheliceral *Al-ptc* stripes in a region taken to be the ocular segment (Figure 1D-E). *Al-ptc* is also expressed in a broad growth zone, possibly populated with undifferentiated segmental tissue (Figure 1B-C´; F). This growth zone is bifurcated in the ventral midline, possibly due to the presence of neuroectodermal tissue of the ventral sulcus (Figure 1D-D´).

*Al-ptc* expression in later stages followed previously reported expression patterns of the *A. longisetosus* orthologues of *hh* and *en*[22] by which the first opisthosomal segment appears initially, followed by the appearance of the segment bearing the anlagen of the fourth pair of walking legs, which is then followed by the appearance of the final second opisthosomal segment. Following the early *Al-ptc* expression in the early germ-band (Figure 1A-F), *Al-ptc* expression was expressed in stripes of expression in the cheliceral and pedipalpal segments as well as the segments of the first three pairs of walking legs (Figure 1G-H´); however, the ‘older’ segments, that is, all prosomal segments excluding the third walking leg segments, had a more pronounced stripe of *Al-ptc* expression in the middle of the segment (Figure 1G), and the posterior stripes were undetected. However, this may be an artifact of our methodology, as in late-stage *D. melanogaster* embryos, the anterior *ptc* stripe of expression is much more pronounced than the posterior stripe [26]. At this stage, no opisthosomal expression of *Al-ptc* was observed, with the posterior-most expression in the posterior limit of the anlagen of the third pair of walking legs (Figure 1H-H´). Following this stage, a broad single stripe of *Al-ptc* expression was observed in the region of the first opisthosomal segment (O1) (Figure 1I-J), similar to the patterns of *Al-hh* and *Al-en* observed in [22]. Also in the same manner as *Al-hh* and *Al-en* expression, a stripe of *Al-ptc* appeared anterior to the O1 stripe in the following stage (Figure 1K). Subsequently, the broad stripe of *Al-ptc* expression in O1 split into two stripes (Figure 1L), likely to facilitate *Al-en* expression, as has been observed in a myriapod [24], a fly [26] and a spider [25]. Following this stage, *Al-ptc* was expressed in a broad stripe demarking the second opisthosomal segment. Also at this stage, the *Al-ptc* expression in the fourth walking leg segment remained in a single stripe (Figure 1M). Whether this is due to the resolution of our images or due to a different patterning mechanism needs to be explored further. In later stages, in which the opisthosoma began to move more anterior forming the caudal bend (see [22] for morphological movements), *Al-ptc* expression in the fourth walking leg segment was reduced, with two broad stripes remaining in the opisthosoma demarking the first and second opisthosomal segments, respectively (Figure 1N-O). Observations of expression patterns are complicated in later stages due to the formation of the caudal bend. Therefore, it is unknown when the *Al-ptc* stripe of the second opisthosomal segment splits nor is it known when the *Al-ptc* stripe of the fourth walking leg segment splits. Further study into these questions using laser-scanning fluorescent confocal microscopy needs to be conducted, as *ptc* genes in other arthropod species are expressed in single-cell wide domains [24-26], and the non-fluorescent detection methods prove problematic in ascertaining the small domains in *A. longisetosus* (personal observations).

### *Ultrabithorax* (*Al-Ubx*) expression

**Figure 2:**
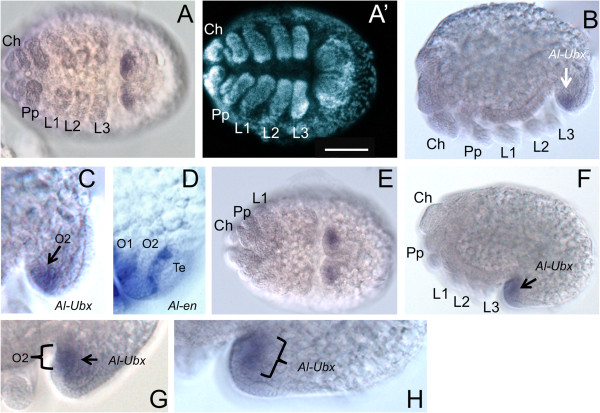
***Al-Ultrabithorax*(*Al-Ubx*) expression.** All embryos are oriented with the anterior pole towards the left. **(A)** The earliest expression of *Al-Ubx* is in a small domain in the opisthosoma. **(A')** DAPI image of the same embryo as in **A**; scale bar is 50 μm. **(B)** Lateral view of the same embryo in **A** showing the small domain of *Al-Ubx* expression in the opisthosoma. **(C)** Close-up of the opisthosoma of the embryo in **B** showing *Al-Ubx* expression in O2. **(D)***Al-en* expression in the opisthosoma used for comparison with *Al-Ubx* expression at the same stage, showing *Al-Ubx* expression only in O2, and its absence in O1 and in the Te. **(E)** Ventral view of a later-stage embryo showing that *Al-Ubx* expression is retained in the small opisthosomal domain. **(F)** Lateral view of the same embryo as in **E**. **(G)** Close-up of the opisthosoma at a stage between those shown in **B** and **F** to illustrate the position of *Al-Ubx* expression in the O2 segment during the formation of the caudal bend. **(H)** Close up of the opisthosomal expression of *Al-Ubx* shown in **F**. Ch, chelicerae; DAPI, 4′,6-diamidino-2-phenylindole; L1-L3, first-third walking legs; O1-O2, first and second opisthosomal segment; Pp, pedipalps; Te, telson.

The single *A. longisetosus Ultrabithorax* orthologue (*Al-Ubx*) was expressed only during the later parts of opisthosomal segmentation (Figure 2). At the earliest stage of *Al-Ubx* expression, *Al-Ubx* is expressed in a small ventral domain that coincides with the boundaries of the second opisthosomal segment delineated by the expression patterns of *Al-ptc* (Figure 1K-O) and *Al-en* (Figure 2A-D). In subsequent stages, following the completion of the formation of the caudal bend, *Al-Ubx* expression remained in this small domain (Figure 2E-H). This expression domain initially looked broader in comparison to earlier stages (compare Figure 2A to 2E). However, under close inspection, this is due to the ‘rolling over’ of the opisthosoma during the formation of the caudal bend (compare Figure 2C, G and H). Therefore, the expression of *Al-Ubx* seen in Figure 2E-F and H is being viewed through posterior tissue that has folded over the *Al-Ubx* expressing cells (see [22] for a review on the morphogenesis of the caudal bend). Thus, the above data suggest that *Al-Ubx* is expressed only in the second opisthosomal segment. These data, in conjunction with the segmentation gene data, suggest that the segments of the opisthosoma reduce in size in later stages.

### *Abdominal-B* (*Al-Abd-B*) expression

**Figure 3:**
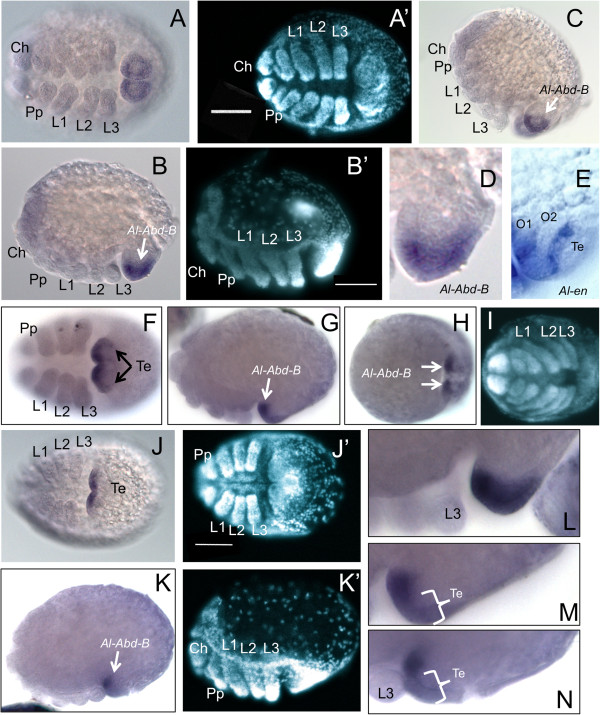
***Al-Abdominal-B*(*Al-Abd-B*) expression.** Embryos are oriented with the anterior to the left unless otherwise noted. **(A)** Ventral view of early *Al-Abd-B* expression. **(A)** DAPI image of the embryo in **A**. **(B)** Lateral view of the embryo in **A**. **(B´)** DAPI image of the embryo in **B**. **(C)** Ventral-lateral view of the embryo in **A** and **B** with anterior towards the top of the page, showing *Al-Abd-B* in the opisthosoma. **(D)** Close-up image of *Al-Abd-B* expression in the opisthosoma in **B**. **(E)***Al-en* expression in the opisthosoma at the same stage as **E**. **(F)** Ventral view of a later staged embryo showing the clearing of *Al-Abd-B* from the Te. **(G)** Lateral view of the embryo in **J**. **(H)** Anterior-ventral view of the embryo in **F** and **G** showing strong *Al-Abd-B* expression in the anterior opisthosoma (arrows). **(I)** DAPI image of the embryo in **H**. **(J)** Ventral view of a later-stage embryo showing that *Al-Abd-B* expression has cleared from the Te. **(J´)** DAPI image of the embryo in **J**. **(K)** Lateral view of the embryo in **J** showing the restricted ‘spot’ of *Al-Abd-B* expression in the opisthosoma. **(K´)** DAPI image of the embryo in **K**. **(L)** Close-up of *Al-Abd-B* in the opisthosoma of an embryo approximately intermediate of **B** and **G**, showing earlier, broad *Al-Abd-B* expression. **(M)** Close-up of the opisthosoma showing *Al-Abd-B* in the same embryo in **G**. As the caudal bend progresses, *Al-Abd-B* expression clears from the Te and becomes restricted to O2. **(N)** Close up of *Al-Abd-B* in the opisthosoma of the embryo in **K** showing the further restriction of *Al-Abd-B* . Scale bars in **A´**, **B´**, **J´** and **K´** are 50 μm. See text for details. Ch, chelicerae; DAPI, 4′,6-diamidino-2-phenylindole; L1-L3, first through third walking legs; O1-O2, first and second opisthosomal segment; Pp, pedipalps; Te, telson.

The *A. longisetosus Abdominal-B* orthologue (*Al-Abd-B*) was also expressed only in later stages in the opisthosoma (Figure 3). In comparison to *Al-Ubx*, *Al-Abd-B* was initially expressed in a broader ventral domain. This domain of expression coincides with the boundaries of the second opisthosomal segment as well as the unsegmented telson (Figure 3A-E and L). In later stages, following the completion of the formation of the caudal bend, *Al-Abd-B* is expressed in a much smaller domain and is expressed weakly in the telson (compare Figure 3A to 3F). Also at this stage, the darker more anterior expression pattern now sits at the anterior-pointing region of the opisthosoma and appears to be situated in the same segmental domain (that is, the second opisthosomal segment) that *Al-Ubx* is expressed in at this stage (Figure 3G-I; compare Figure 2F and H to Figure 3G and M). In subsequent stages, *Al-Abd-B* is restricted to the second opisthosomal segment and all expression has been removed from the telson (Figure 3K-K´ and N). These data indicate that, like *Al-Ubx*, *Al-Abd-B* is expressed only in the second opisthosomal segment; however, *Al-Abd-B* is initially expressed in the telson until the later stages of the formation of the caudal bend.

## Discussion

### *Al-ptc* expression provides evidence that the *wg/en* segmentation pathway is conserved in mites

In the fly *D. melanogaster*, terminal segmental boundaries are generated by the Engrailed/Wingless auto regulatory loop [28], whereby *en* expressing cells activate *hh* expression and signaling. Hedgehog signaling proteins bind to the Ptc receptor proteins on the anterior adjacent cells to activate the expression of *wingless*, which encodes a signaling molecule that in turn binds to the Frizzled-2 receptor in the *en* expressing cells, thereby stabilizing *en* expression. This signaling pathway has been shown to be conserved in a number of other arthropods (see [29] for review). *patched* expression has been observed in three arthropod groups, in the fly *D. melanogaster*[26], the millipede *G. marginata*[24] and the spider *Parasteatoda tepidariorum*[25]. In *D. melanogaster*, *ptc* is initially ubiquitously expressed in the blastoderm. In later stages, *ptc* is expressed in a segmental manner in cells abutting *en* expressing cells, via the repression of *ptc* by *en*. In subsequent stages, *ptc* is repressed by an unknown factor, resulting in two thin stripes of *ptc* expression per segment with *en* expressing cells in the middle of these two stripes [26]. This two-striped expression pattern of *ptc* surrounding a stripe of *en* expression is also seen in the ventral germ band of the millipede *G. marginata*[24] and also in the developing segments in the spider *P. tepidariorum*[25]. In all three of these species, *ptc* is initally expressed in broad stripes prior to the splitting into two stripes per segment. The data presented for *Al-ptc* expression (Figure 1) suggests that this mode of *ptc* expression is conserved throughout the arthropods. Previous data on *en* and *hh* expression in *A. longisetosus* indicate that the En/Wg signaling loop acts in *A. longisetosus* to pattern terminal segmental boundaries [22,30]. However, the expression patterns of other components of this pathway (for example, *wingless*, *cubitus interruptus* and *Notum*) are needed to confirm this.

### *Al-Ubx* is expressed in a single segment: a novel observation in an arthropod

The above data illustrate that in the mite *A. longisetosus*, *Ubx* is expressed in a single segment. The conserved role of Hox genes in specifying segments in arthropods suggests that *Al-Ubx* specifies the identity to a single segment in the opisthosoma. This is a novel observation in an arthropod in that in all observed arthropod species, *Ubx* is expressed in several developing posterior segments. In insects, *Ubx* specifies the abdominal segments, and in some lineages, *Ubx* is expressed in the second and/or third thoracic segments (for example, [31-35]). In crustaceans, *Ubx* is also expressed in multiple posterior developing segments [36-38], as is *Ubx* in myriapods [39-42].

Direct expression patterns of *Ubx* in chelicerates have been observed in the spiders (Araneae) *P. tepidariorum* and *Cupiennius salei*, and also in the harvestman *Phalangium opilio*. In *P. tepidariorum*, the single identified *Ubx* orthologue is expressed in the second opisthosomal segment (O2) through the remaining posterior segments [21]. *C. salei* has two orthologues of *Ubx*, in which *Ubx-1* is expressed from the anterior portion of O2 through the remaining posterior segments. *Ubx-2* is expressed from the posterior half of O2 through the remaining posterior opisthosomal segments [16]. *Ubx* expression in *P. opilio* is similar to spider expression patterns, where its anterior border of expression also lies in O2 [18]. Popadić and Nagy (2001) observed expression patterns of the Hox genes *Ubx* and *abd-A* simultaneously using the UbdA antibody in the scorpion *Paruroctonus mesaensis* and the horseshoe crab *Limulus polyphemus*. The detection of this antibody showed that *Ubx* has an early expression boundary in O2 in both species, which later moves anteriorly to be expressed in O1. These data indicate that the ancestral chelicerate expression boundary of *Ubx* lies in the second opisthosomal, or genital, segment. However, the UbdA data should be interpreted with caution, as this antibody detects both *Ubx* and *abd-A* expression.

The *Al-Ubx* expression data indicate that this gene patterns a single segment, the second opisthosomal segment (Figure 2). This adds support to the hypothesis that the second opisthsosomal segment was the ancestral *Ubx* anterior expression boundary in chelicerates. We, therefore, maintain that the first and second opisthosomal segments are retained in *A. longisetosus* due to this anterior expression boundary of *Al-Ubx*. However, unlike the *Ubx* expression patterns observed in other chelicerates, *Al-Ubx* was not observed to extend anteriorly or posteriorly in later stages.

### *Al-Abd-B* is expressed in a single segment; a novel observation in an arthropod

The expression data for *Al-Abd-B* also indicate that this gene is expressed in a single segment as well as in an early domain in the unsegmented telson (Figure 3). This is also a unique observation for arthropods in that in many studied arthropods, *Abd-B* patterns multiple posterior segments during development (see [6] for review). In the fly *D. melanogaster*, *Abd-B* functions to specify the fourth through the eighth abdominal segments via the expression of the m and r *Abd-B* isoforms [43]. Abd-B also has a role in specifying the genital region of *D. melanogaster* embryos [44,45]. In the beetle *Tribolium castaneum, Abd-B* also acts to specify the posterior ninth and tenth abdominal segments [46]. In the grasshopper *Schistocerca gregaria*, *Abd-B* is expressed in the eighth through the eleventh abdominal segments, as well as in the genital region [47]. In the thysanuran *Thermobia domestica*, *Abd-B* is expressed in the eight through the tenth abdominal segments [48]. In the milkweed bug *Oncopeltus fasciatus*, *Abd-B* also has a genital-specifying role [49]. In crustaceans, *Abd-B* is also expressed in the genital segments [50-52] and extends throughout all five segments of the posterior tagma (the pleon) of the isopod *Porcellio scaber*[51], but remains in the genital region of *Artemia franciscana*[50]*.* In the cirripede *Sacculina carcini*, *Abd-B* is expressed throughout the thorax and also in the vestigial abdomen [52]. For myriapods, *Abd-B* is expressed from the second leg-bearing segment posteriorly to the telson in the centipede *Lithobius atkinsoni*[42] and is expressed only in the posterior growth zone and the anal valves in the millipede *Glomeris marginata*; however, as *G. marginata* undergoes anamorphic growth (that is, more segments are added in post-embryonic stages) *Abd-B* may be expressed in these posterior segments at later stages [39]. These data, therefore, indicate that *Abd-B* had an ancestral role in patterning multiple posterior segments in arthropods, as well as a possible ancestral role in specifying the genital region.

In the chelicerates, *Abd-B* expression has also been observed in the spider *C. salei* and the harvestman *P. opilio*. In *C. salei*, *Abd-B* has a later expression domain in the cells of the future genital opening of O2, consistent with the hypothesis that *Abd-B* had a role of genital patterning in the last common ancestor of arthropods, as well as with the hypothesis that *Abd-B* had a role in patterning the genital region in the last common ancestor of the protostomes and the deuterostomes ([19] and references therein). Our observations show that *Al-Abd-B* is expressed in what we interpret as the second opisthosoma segment, due to the expression of *Al-Ubx* in this segment (see above). However, we are unable to assess when and where the genital rudiments of *A. longisetosus* form, which would verify that the segment expresses both *Al-Ubx* and *Al-Abd-B*. This may be due to our methodology, or due to the complex morphogenesis of the caudal bend during the growth of the opisthosoma. An earlier expression pattern for *Al-Abd-B* was also not observed in stages earlier than those shown in Figure 3A-D. We, therefore, maintain our interpretation that the two segments observed in the *A. longisetosus* opisthosoma are the first and second opisthosomal segments.

### *abdominal-A* loss and posterior segmental reductions in arthropods

Like the spider mite *Tetranychus urticae*[27], *A. longisetosus* is likely missing the Hox gene *abd-A*, as Hox cluster sequencing and PCR surveys have yielded no *abd-A* orthologue (RH Thomas, unpublished results). *T. urticae* and *A. longisetosus* also display a two-segmental pattern of *en* expression in their opisthosomas, indicating that a loss of *abd-A* and the reduction of the opisthosoma occurred at the base of the acariform mite lineage [22,27,30]. In all arthropods that retain an *abd-A* orthologue in their genome, *abd-A* is expressed in posterior regions overlapping *Ubx* and *Abd-B* expression (see [6] for review). In the cirripede crustacean *S. carcini*, the abdominal segments are never fully developed in the adult. However, an *en* expression studied indicated that *S. carcini* patterned five abdominal segments in the developing vestigial abdomen, which are later removed following metamorphosis [53]. Interestingly, hybridization experiments failed to find an expression domain for *abd-A* in any region during the development of *S. carcini*[52], and a subsequent cytogenic analysis found no putative *abd-A* orthologues in the *S. carcini* Hox cluster [54]. Pycnogonids (sea spiders) also have a reduced posterior tagma. A PCR survey of Hox genes failed to find an *abd-A* orthologue in the pycnogonid *Endeis spinosa*; it also found that the *abd-A* orthologue in the pycnogonid *Nymphon gracile* had an unusual degree of sequence divergence in its homeodomain, possibly due to relaxed selection [55].

The reduction of posterior segmentation and the absence of an *abd-A* orthologue in these three disparate arthropod groups display a surprising convergent correlation. There may be some trend in arthropod evolution whereby redundant Hox gene functions in segments expressing multiple Hox genes cause the loss of selective pressure retaining one of the redundant genes in the genome. This selective pressure may also be reduced in arthropod lineages in which the posterior segments are reduced. Also, if selection is acting to reduce posterior segments, selection should act to retain only those posteriorly expressed Hox genes with multiple, non-redundant functions that are also highly pleiotropic. *Ubx* has functions that overlap with those of *abd-A* in arthropods, (for example, [56,57]). Therefore, *abd-A* may have been a target of reduced selection to maintain its presence on the genomes of different arthropod groups during evolution.

As Hox genes act to specify the identities of segments in arthropods, it should follow that the loss of Hox genes (that is, *abd-A*) followed the loss of segments. In *A. longisetosus,* it would seem likely that segmental loss was facilitated via repressing the production of posterior segments arising from the posterior growth zone, rather than eliminating anterior segments. However, this is complicated as *A. longisetosus* patterns posterior segments in a manner that is not currently well-understood, in that it follows an anachronistic delineation pattern (that is, the later appearance of the L4 segment following the delineation of the first opisthosomal segment). Therefore, comparative functional studies are needed to answer these questions surrounding the loss of arthropod Hox genes and posterior segments.

### Hox genes and chelicerate tagmosis

**Figure 4:**
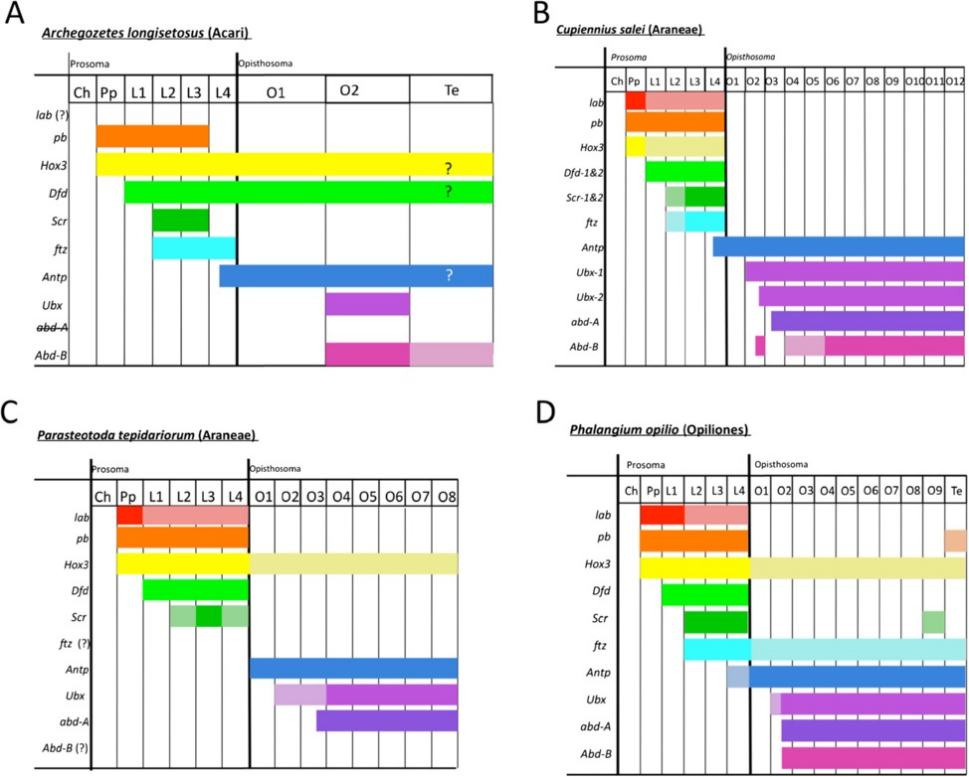
**Representation of the known Hox expression domains in the segments of*A. longisetosus*, the spiders*Cupiennius salei*and*Parasteatoda tepidariorum*, and the harvestman*Phalangium opilio*.(A)** The known Hox expression domains in the mite *A. longisetosus*. Expression data for *pb*, *Dfd*, *Scr*, and *Antp* from [30]. Expression data for *Hox3* from [59] and *Ftz* expression from [60]. Question marks for *Hox3, Dfd* and *Antp* denote the unknown late-expression patterns in the Te (see text). **(B)** The known Hox gene expression domains for the spider *C. salei*. *lab, Antp, Ubx-1* and *2* and *abd-A* expression from [16]; *pb, Dfd*-*1* and *2* and *Scr*-*1* and *2* expression from [61]. Note that *Scr-2* expression was not observed in L2; *Hox3* expression from [62]; *Ftz* from [63]; *Abd-B* from [20]. **(C)** The known Hox expression domains for the spider *P. tepidariorum. lab, Dfd, Scr, Antp, Ubx*, and *abd-A* modified from [21] and *pb* and *Hox3* from [58]. **(D)** The known Hox expression domains for the harvestman *P. opilio*, adapted from [18]. Shaded bars indicate weak expression; however, the shaded bar for *Abd- B* expression in *A. longisetosus* denotes its early expression in the Te and subsequent clearing from this tissue. *abd-A abdominal-A*; *abd-B, Abdominal-B; Antp, Antennapedia;* Ch, chelicerae; *Dfd, Deformed; ftz, fushi-tarazu;* L1-L4, first through the fourth walking legs; *lab, labial;* O1-O12, first through twelfth opisthosomal segments; *pb, proboscepedia;* Pp, pedipalps; *Scr, Sex combs reduced;* Te, telson; *Ubx, Ultrabithorax.*

The co-expression of arthropod Hox genes has been shown to correlate with tagmatization, or the fusion of body segments to form distinct morphological units along the antero-posterior axis. Previous work has shown that the expression of Hox genes in spiders correlates with the prosomal and opisthosomal boundaries, with the genes *labial* (*lab*), *proboscepedia* (*pb*), *Hox3*, *Deformed* (*Dfd*), and *Sex combs reduced* (*Scr*) being expressed in the prosoma, and the remaining Hox genes, *Antennapedia* (*Antp*), *Ubx*, *abd-A*, and *Abd-B*, being expressed predominately in the opisthosoma. *Hox3* expression in *P. tepidariorum* and *P. opilio* are notable exceptions, being expressed strongly in the pedipalpal and walking leg segments, and weakly throughout the opisthosoma [18,58](Figure 4C-D). Also, in *P. opilio*, *ftz* is weakly expressed throughout the opisthosoma, and *pb* and *Scr* have weak expression domains in the telson and ninth opisthosomal segments respectively [18] (Figure 4D). *Antp* also breaks the prosoma-opisthosoma boundary in *A. longisetosus*[30] and *C. salei*[16] with both having an anterior boundary in the posterior portion of the fourth walking leg segment. This is also seen in *P. opilio* in which *Antp* is expressed in the entire fourth walking leg segment [18] (Figure 4).

In comparison to other chelicerates, *A. longisetosus* differs in its utilization of Hox genes to pattern segments. Most notably is the single segment of expression of *Ubx* and *Abd-B* in the opisthosoma (Figures 2 and 3). Also of note is that *Hox3* and *Dfd* are expressed in both the prosoma and opisthosoma in *A. longisetosus* (Figure 4A), breaking the tagmatic boundary rule. This observation coupled with the weak expression of *Hox3* observed in the opisthosoma of *P. tepidariorum* and *P. opilio* may indicate a conserved role of *Hox3* in chelicerates, with the expression patterns in *C. salei* being derived. The remaining Hox genes are expressed in a similar manner to the *P. opilio*, *C. salei* and *P. tepidariorum*, and do not seem to correlate with the borders of the pseudo-tagmata.

The absence of *abd-A* in *A. longisetosus* may be tied to the expression patterns of *Hox3* and *Dfd* in the opisthosoma, as they indicate that extra Hox input is needed in these segments. However, functional studies of these genes in *A. longisetosus* are needed before this can be confirmed. Comparative functional studies of segmentation and posterior Hox gene expression in mites will be necessary to reveal the selection pressures, that is, a reduction in segmentation, miniaturizations, and so on that have led to their loss of *abd-A*.

Studies are also needed to elucidate how *Hox3, Dfd* and *Antp* expression patterns change in the opisthosoma throughout development. Telford and Thomas [59] show *Al-Hox3* expression in the opisthosoma; however, this is at an early stage and its late-stage expression patterns in the opisthosoma are unknown. Likewise, Telford and Thomas [30] show *Al-Dfd* expression throughout the opisthosoma; however, its late-stage expression in the telson is unclear. This study also highlights *Al-Antp* expression in an embryo of the same age as those shown in Figures 2B and 3B. However, whether *Al-Antp* clears from the telson on later stages is also unclear. Therefore, further study is need into the interactions and dynamic expression patterns of Hox genes in the mite opisthosoma.

## Conclusions

The above data illustrate the reduced Hox gene input in the opisthosoma of the mite *A. longisetosus* by examining the expression of the *A. longisetosus* orthologues of *Ubx* and *Abd-B*. These two Hox genes are restricted in later stages to the same opisthosomal segment, namely the second opisthosomal segment. The reduced segmental composition in the *A. longisetosus* opisthosoma [22], coupled with the confirmed absence of *abd-A* in one acariform mite [27] and a likely loss of *abd-A* in *A. longisetosus* (RH Thomas, unpublished results), calls for further study into the evolution of the mite opisthosoma.

## Abbreviations

abd-A: *Abdominal-A*; abd-B: *Abdominal-B*; Al-Abd-B: *Archegozetes longisetosus Abdominal-B*; Antp: *Antennapedia*; Al-Ubx: *Archegozetes longisetosus Ultrabithorax*; Al-en: *Archegozetes longisetosus engrailed*; bp: Base pair; Ch: Chelicerae; DAPI: 4′,6-diamidino-2-phenylindole; Dfd: *Deformed*; ftz: *fushi-tarazu*; GZ: Growth zone; L1: First walking leg; L2: Second walking leg; L3: Third walking leg; L4: Anlagen of the fourth pair of walking legs; Oc: Ocular segment; O1: First opisthosomal segment; O2: Second opisthosomal segment; PBS: Phosphate-buffered saline; PCR: Polymerase chain reaction; PL3: Posterior *Al-ptc* stripe of the third walking leg; pb: *Proboscepedia*; Pp: Pedipalp; Pr: Proctodaeum; RACE: Rapid amplification of cDNA ends; Scr: *Sex combs reduced*; Te: Telson; Ubx: *Ultrabithorax*; UTR: Untranslated region; VS: Ventral sulcus.

## Competing interests

The authors declare that they have no competing interests.

## Authors’ contributions

RHT and AAB conceived the study. AAB cloned all of the genes followed in this study, and performed the *in situ* hybridizations, and sequence analyses. Both authors participated in writing the manuscript, and both have discussed and approved the final version of the manuscript.

## Supplementary Material

Additional file 1: Figure S1 Multiple sequence alignments of the deduced amino acid sequences of *Al-Ubx, Al-Abd-B* and *Al-ptc against selected orthologues*. (A) Multiple sequence alignment of the deduced *Al-ptc* Eukaryotic Sterol Transporter (EST) Family domain against Ptc orthologue protein sequences. Note: Only *I. scapularis* Ptc-2 was retrieved in a genome blastx against *Al*-*ptc*. (B) Multiple sequence alignment of the deduced *Al-Ubx* homeodomain and UbdA motif aligned against Ubx orthologues. (C) Multiple sequence alignment of the deduced *A-l-Abd-B* homeodomain aligned against other *Abd-B* orthologue protein sequences. All sequences retrieved from GenBank except for *T*. *urticae* and *I. scapularis* sequences which were retrieved from bioinformatics.psb and VectorBase, respectively. Species abbreviations are *T. urticae* (*Tetranychus urticae*, Chelicerata, Acari); *I. scapularis* (*Ixodes scapularis*, Chelicerata, Acari); *P. tepidariorum* (*Parasteatoda tepidariorum,* Chelicerata, Araneae); *C. salei* (*Cupiennius salei,* Chelicerata, Araneae); *E. spinosa* (*Endeis spinosa,* Chelicerata, Pycnogonida); *G. marginata* (*Glomeris marginata,* Myriapoda, Diplopoda); *S. maritima* (*Strigamia maritima,* Myriapoda, Chilopoda); *A. franciscana* (*Artemia franciscana,* Crustacea, Anostraca); *D. pulex* (*Daphnia pulex,* Crustacea, Cladocera); *S. carcini* (*Sacculina carcini,* Crustacea, Cirripedia); *P. scaber (Porcellio scaber,* Crustacea, Isopoda); *C. floridanus* (*Camponotus floridanus,* Hexapoda, Hymenoptera); *A. echinatior* (*Acromyrmex echinatior,* Hexapoda, Hymenoptera); *H. saltator* (*Harpegnathos saltator,* Hexapoda, Hymenoptera); *N. vitripennis* (*Nasonia vitripennis,* Hexapoda, Hymenoptera); *B. terrestris* (*Bombus terrestris,* Hexapoda, Hymenoptera); *C. quinquefasciatus* (*Culex quinquefasciatus,* Hexapoda, Diptera); *D. melanogaster* (*Drosophila melanogaster*, Hexapoda, Diptera); *A. gambiae* (*Anopheles gambiae,* Hexapoda, Diptera); *B. betularia* (*Biston betularia,* Hexapoda, Lepidoptera); *F. candida* (*Folsomia candida*, Hexapoda, Collembola); *A. kaputensis* (*Akanthokara kaputensis,* Onychophora). Click here for file
