# Supplementary figures and images for: Posterior Hox gene reduction in an arthropod: Ultrabithorax and Abdominal-B are expressed in a single segment in the mite Archegozetes longisetosus
Source: EvoDevo. 2013 Aug 30;4:23. doi: 10.1186/2041-9139-4-23 (PMC3766265; doi:10.1186/2041-9139-4-23)

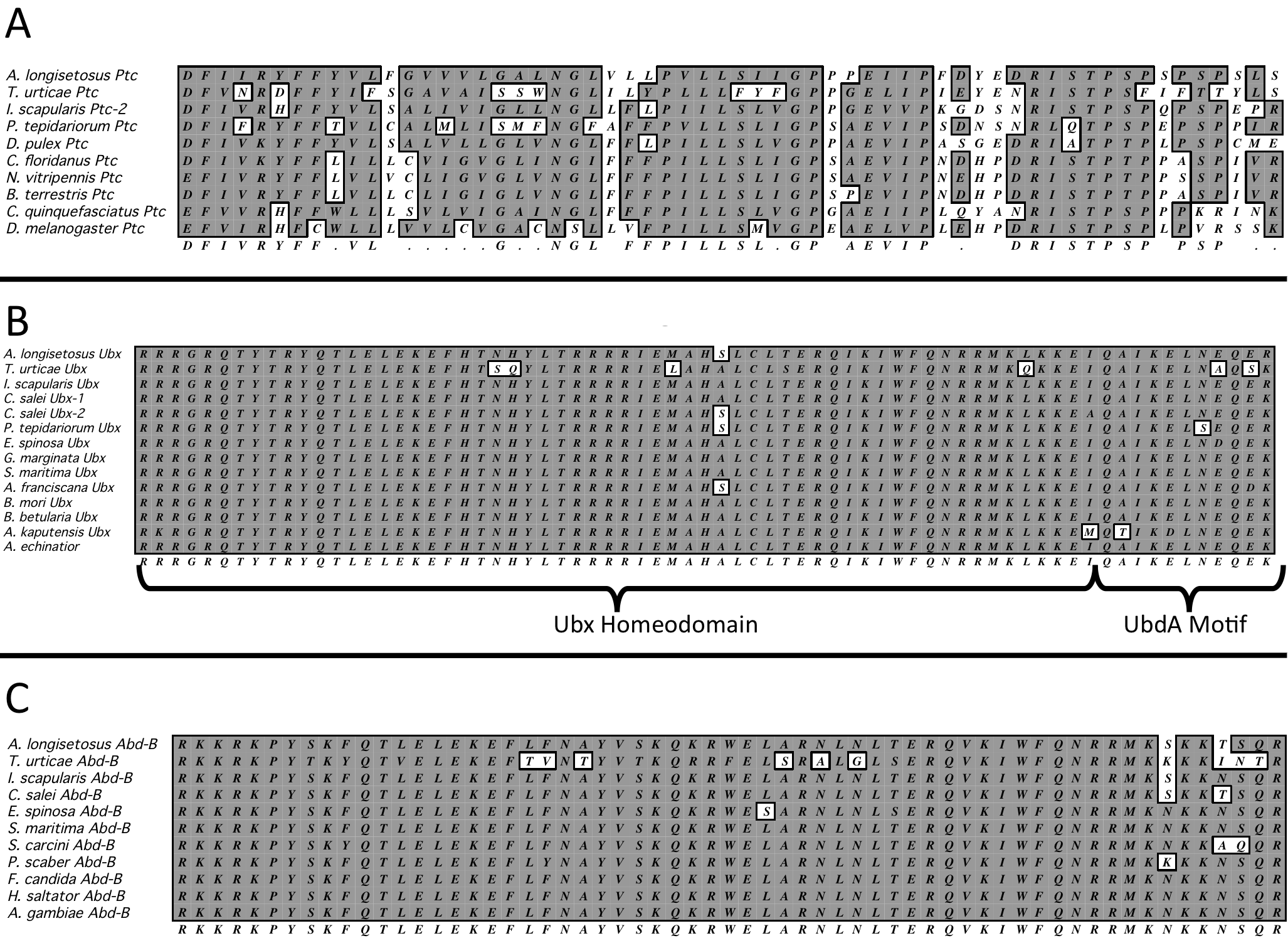

Supplement: Additional file 1: Figure S1 — Multiple sequence alignments of the deduced amino acid sequences of Al-Ubx, Al-Abd-B and Al-ptc against selected orthologues. (A) Multiple sequence alignment of the deduced Al-ptc Eukaryotic Sterol Transporter (EST) Family domain against Ptc orthologue protein sequences. Note: Only I. scapularis Ptc-2 was retrieved in a genome blastx against Al-ptc. (B) Multiple sequence alignment of the deduced Al-Ubx homeodomain and UbdA motif aligned against Ubx orthologues. (C) Multiple sequence alignment of the deduced A-l-Abd-B homeodomain aligned against other Abd-B orthologue protein sequences. All sequences retrieved from GenBank except for T. urticae and I. scapularis sequences which were retrieved from bioinformatics.psb and VectorBase, respectively. Species abbreviations are T. urticae (Tetranychus urticae, Chelicerata, Acari); I. scapularis (Ixodes scapularis, Chelicerata, Acari); P. tepidariorum (Parasteatoda tepidariorum, Chelicerata, Araneae); C. salei (Cupiennius salei, Chelicerata, Araneae); E. spinosa (Endeis spinosa, Chelicerata, Pycnogonida); G. marginata (Glomeris marginata, Myriapoda, Diplopoda); S. maritima (Strigamia maritima, Myriapoda, Chilopoda); A. franciscana (Artemia franciscana, Crustacea, Anostraca); D. pulex (Daphnia pulex, Crustacea, Cladocera); S. carcini (Sacculina carcini, Crustacea, Cirripedia); P. scaber (Porcellio scaber, Crustacea, Isopoda); C. floridanus (Camponotus floridanus, Hexapoda, Hymenoptera); A. echinatior (Acromyrmex echinatior, Hexapoda, Hymenoptera); H. saltator (Harpegnathos saltator, Hexapoda, Hymenoptera); N. vitripennis (Nasonia vitripennis, Hexapoda, Hymenoptera); B. terrestris (Bombus terrestris, Hexapoda, Hymenoptera); C. quinquefasciatus (Culex quinquefasciatus, Hexapoda, Diptera); D. melanogaster (Drosophila melanogaster, Hexapoda, Diptera); A. gambiae (Anopheles gambiae, Hexapoda, Diptera); B. betularia (Biston betularia, Hexapoda, Lepidoptera); F. candida (Folsomia candida, Hexapoda, Collembola); A. kaputensis (Akanth [file 2041-9139-4-23-S1.png]
